# Genome-wide review of transcriptional complexity in mouse protein kinases and phosphatases

**DOI:** 10.1186/gb-2006-7-1-r5

**Published:** 2006-01-26

**Authors:** Alistair RR Forrest, Darrin F Taylor, Mark L Crowe, Alistair M Chalk, Nic J Waddell, Gabriel Kolle, Geoffrey J Faulkner, Rimantas Kodzius, Shintaro Katayama, Christine Wells, Chikatoshi Kai, Jun Kawai, Piero Carninci, Yoshihide Hayashizaki, Sean M Grimmond

**Affiliations:** 1 Institute for Molecular Bioscience and ARC Centre in Bioinformatics, University of Queensland, Brisbane, QLD 4072, Australia; 2 Queensland Institute for Medical Research, PO Royal Brisbane Hospital, Brisbane, QLD 4029, Australia; 3 Center for Genomics and Bioinformatics, Karolinska Institutet, S-171 77 Stockholm, Sweden; 4 Genome Exploration Research Group (Genome Network Project Core Group), RIKEN Genomic Sciences Center (GSC), RIKEN Yokohama Institute, Yokohama, Kanagawa, 230-0045, Japan; 5 The Eskitis Institute for Cell and Molecular Therapies, Griffith University, QLD 4111, Australia; 6 Genome Science Laboratory, Discovery Research Institute, RIKEN Wako Institute, Wako, Saitama, 351-0198, Japan

## Abstract

**Background:**

Alternative transcripts of protein kinases and protein phosphatases are known to encode peptides with altered substrate affinities, subcellular localizations, and activities. We undertook a systematic study to catalog the variant transcripts of every protein kinase-like and phosphatase-like locus of mouse .

**Results:**

By reviewing all available transcript evidence, we found that at least 75% of kinase and phosphatase loci in mouse generate alternative splice forms, and that 44% of these loci have well supported alternative 5' exons. In a further analysis of full-length cDNAs, we identified 69% of loci as generating more than one peptide isoform. The 1,469 peptide isoforms generated from these loci correspond to 1,080 unique Interpro domain combinations, many of which lack catalytic or interaction domains. We also report on the existence of likely dominant negative forms for many of the receptor kinases and phosphatases, including some 26 secreted decoys (seven known and 19 novel: Alk, Csf1r, Egfr, Epha1, 3, 5,7 and 10, Ephb1, Flt1, Flt3, Insr, Insrr, Kdr, Met, Ptk7, Ptprc, Ptprd, Ptprg, Ptprl, Ptprn, Ptprn2, Ptpro, Ptprr, Ptprs, and Ptprz1) and 13 transmembrane forms (four known and nine novel: Axl, Bmpr1a, Csf1r, Epha4, 5, 6 and 7, Ntrk2, Ntrk3, Pdgfra, Ptprk, Ptprm, Ptpru). Finally, by mining public gene expression data (MPSS and microarrays), we confirmed tissue-specific expression of ten of the novel isoforms.

**Conclusion:**

These findings suggest that alternative transcripts of protein kinases and phosphatases are produced that encode different domain structures, and that these variants are likely to play important roles in phosphorylation-dependent signaling pathways.

## Abstract 2

A systematic study of the transcript variants of all protein kinase- and phosphatase-like loci in mouse shows that at least 75% of them generate alternative transcripts, many of which encode different domain structures.

## Background

The completion of the human and mouse genome sequences has provided the means to study the total mammalian gene complement *in silico *[1,2]. Subsequently, global transcription surveys have been used to provide a more accurate estimate of the transcribed regions of the genome and the structure of genes. According to these studies, 40-60% of loci in higher eukaryotes are predicted to generate alternative transcripts via the use of alternative splice junctions, transcription start sites, and transcription termination sites [3-6].

By generating alternative transcripts, the functional output of the locus can be increased. Alternative transcripts can encode variant peptides with altered stability, localization, and activity [7,8]. They can change the 5' and 3' untranslated regions of the message, which are known to be important in translation efficiency and mRNA stability [9-11], and in the case of alternative promoters they allow a gene to be switched on under multiple transcriptional controls [12,13].

One area in which the impact of alternative transcripts has not been fully assessed is in systems biology. In recent years workers have moved toward modeling entire biologic systems, including signal transduction pathways and transcriptional networks [14]. Key tasks are to define the components of the system in question and then to determine how they interact. The role played by alternative transcripts and peptide isoforms generated by regulated transcriptional events in these systems has not been addressed [14,15].

One such system is that regulating protein phosphorylation states. In addition to regulatory subunits, inhibitors, activators, and scaffolds, protein phosphorylation is regulated by two classes of enzymes: the protein kinases, which attach phosphate groups; and the protein phosphatases, which remove them. Reports of alternative isoforms of these proteins are common and for some loci such as HGK, which contains nine reported alternatively spliced modules, the number of variants themselves is impressive [16]. For these enzymes variants that alter or remove the catalytic domain are known to affect activity and substrate specificity [17,18]. In others, such as the fibroblast growth factor receptors Fgfr1 and 2, restricted expression of splice variants with altered ligand binding domains allow cells to elicit tissue specific responses [19].

To examine the impact of alternative transcripts on this system we undertook a systematic study of the variant transcripts of mouse protein kinase and protein phosphatase loci; we refer to these collectively as the phosphoregulators. To do this we exploited the wealth of mouse full-length cDNA sequences generated by the Functional Annotation of Mouse 3 (FANTOM3) project [20] and all available public mouse cDNA sequences. We report on the frequency of alternative forms, domain content, and the levels of support for each isoform, and we speculate on the role these isoforms are likely to play in the regulation of protein phosphorylation.

## Results

### The kinase-like and phosphatase-like loci of mouse

Before attempting to catalogue the alternative transcripts of mouse protein kinase-like and phosphatase-like loci of mouse, we first reviewed all putative kinases and phosphatases identified in the literature and combined the results with new sequences identified by InterProScan predictions of open reading frames (ORFs) from the FANTOM3, GenBank, and Refseq databases (Sequnces used in the analysis were all those available at September 2004) [20-23].

In 2003 we estimated that there are 561 kinase-like genes in mouse, using the domain predictor InterProScan [21] to identify sequences containing kinase-like motifs in all available cDNA sequences and all ENSEMBL gene predictions [22]. In 2004 an alternative estimate of 540 kinase-like genes was reported [23,24]. We undertook a systematic review of both data sets and now revise the estimate down to 527 kinase-like loci, and there is transcriptional evidence for 522 of these. We removed all false positives introduced by the ProSite kinase domain motif (PSOO107), and duplicates introduced by partial ENSEMBL gene predictions. Similarly, for the phosphatase-like loci of mouse we revised the estimate to 160 loci, and there is transcriptional evidence for 158 of these. We summarize the evidence for each locus in Additional data file 1.

The FANTOM3 data set identified three new kinase-like loci. These are I0C0018M10 (hypothetical protein kinase; GenBank:AK145348), Gm655 (hypothetical serine/threonine kinase; GenBank:AK163219), and a second transcriptionally active copy of the TP53-regulating kinase (Trp53rk; GenBank:AK028411). The kinase-like loci I0C0018M10 and Gm655 appear to represent transcriptionally active pseudogenes with truncated kinase domains. Despite this, the transcripts are not predicted to undergo nonsense mediated decay (NMD), and as such they may still produce truncated kinase-like peptides of unknown biology. The second copy of Trp53rk appears to have arisen from local tandem duplication on chromosome 2. Both copies are supported by expressed sequence tag (EST) and capped analysis of gene expression (CAGE) evidence and have intact ORFs. Although the syntenic copy of Trp53rk (Genbank:AK167662) lies within a region of chromosome 2 that shares the same gene order as a region of human chromosome 20 between the Sl2a10 and Slc13a3 loci, the new locus is adjacent to Arfgef2 locus and is not conserved in human.

### Identifying the transcripts of the phosphoregulator transcriptome

As part of the FANTOM3 project, a transcript clustering algorithm was developed that grouped sequences with shared splice sites, transcription start sites, or transcription termination sites into transcriptional frameworks [20]. These frameworks effectively define the set of cDNA sequences observed for each locus. Using a representative cDNA sequence for each phosphoregulator, we extracted the corresponding framework cluster, the set of all observed cDNA sequences (ESTs and full-length sequences from FANTOM, GenBank, and RefSeq; November 2004), and the genomic mappings for each cDNA (5', 3', and splice junctions). Additionally, high throughput 5' end sequences from CAGE [25] and 5'-3' DiTag sequences (Genomic Sciences Center [20] and gene identification signature [26] DiTag sequences) were also mapped to these framework clusters and used to provide additional support for alternative 5' and 3' ends. The cDNA resources are summarized in Tables 1 and 2.

By combining these cDNA and tag resources, we reviewed the level of support for each transcript. The ORF of each full-length transcript was also assessed to determine whether it encoded a variant peptide and whether the variant had an altered domain structure. These results were compiled into a database and can be viewed online [27]. This web-based interface permits visualization of each locus in its genomic context and provides an annotated view of each transcript with access to peptide and domain predictions (Additional data file 2).

### Alternatively spliced transcripts of the phosphoregulator transcriptome

With all alternative transcripts for the mouse phosphoregulators identified, we then searched for the level of support for each alternative transcription start site, termination site, and splice junction event. For the analysis of splice junctions we clustered pairs of splice donors and acceptors based on their genomic coordinates (Additional data file 3). When a given donor mapped to multiple acceptors, or acceptor to multiple donors, the junction was considered alternative. For an alternative junction to be considered reliable we required there to be two independent cDNA sequences for each alternative (for example, two sequences showing Donor1 spliced to Acceptor1 and two sequences showing Donor1 spliced to Acceptor2). Using these criteria, 75% of the multi-exon phosphoregulator loci appear to undergo alternative splicing. If we consider only single cDNAs as evidence then the frequency increases to 91%. We also compared this with the frequency of alternative splice junction usage in the entire set of transcriptional frameworks (31,541) and a class of loci with a reported high level of alternative splice forms, namely the zinc finger proteins [28]. For these sets, 39% of all multi-exon frameworks and 80% of zinc finger protein encoding frameworks have at least two cDNAs supporting an alternative splice form (53% and 93% for one cDNA; Additional data file 6).

### Alternative transcription initiation and termination of phosphoregulator transcripts

Because of the nature of cDNA synthesis and the possibility of 5' and 3' truncated sequences, we modified the metric used to identify loci with alternative 5' and 3' terminal exons. Alternative initiation and termination were assessed in two steps. First, terminal exon sequences for all multi-exon loci were clustered on the basis of identical first donor sites (for 5' exons) or final acceptor sites (for 3' exons). Secondly, support for transcription start sites (TSS) and transcription termination sites (TTS) within these terminal exons was determined by clustering the terminal 20 bases of 5' and 3' end sequences (cDNA, EST, and tag resources; Table 2) into tag clusters.

By combining these two analyses, tag cluster count was used to provide supporting evidence for each 5' and 3' exon. To identify transcripts with well supported terminal exons, we considered a threshold of five counts to represent reliability. Using this threshold 612 multi-exon loci had well supported 5' terminal exons, and of these 272 (44%) had multiple 5' terminal exons. Similarly, for 3' terminal exons 611 loci had well supported 3' ends, and of these 229 (37%) had multiple 3' terminal exons. Increasing the requirements to a more conservative threshold of 50 tags revealed that 10.7% and 7.3% of these loci used alternative 5' and 3' exons, respectively (Table 3 and Additional data file 4).

In addition, we examined how many of the terminal exons with 50 counts or more had multiple TSS or TTSs within them. Requiring 10 counts to be considered a reliable TSS/TTS, 16% of 5' exons and 47% of 3' exons had more than one reliable TSS/TTS (10 or more counts for each). In the case of the 3' exons, changes in untranslated region length may be functionally relevant or they may just reflect the need for multiple poly-adenylation signals for an inefficient termination process.

### Alternative 5' exon usage

**Figure 1:**
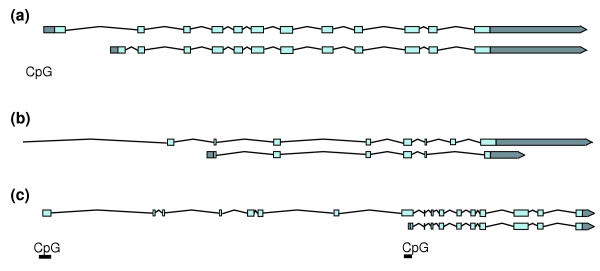
Three types of alternative transcription starts identified in this study. **(a) **ME-Exon: mutually exclusive starting exons (Sgk; GenBank:AK132234 and GenBank:AK086892). **(b) **Intronic: starts within introns that run into the next exon (Egfr; GenBank:AF275367 [longer form] and GenBank:AK087861 [shorter intronic start form]). **(c) **Exonic: starts within exon of longer transcript (Ntrk1; GenBank:AK081588 and GenBank:AK148691; supported by a CpG island and 5'-RACE). 5'-RACE, 5' rapid amplification of cDNA ends.

With an estimate that alternative 5' terminal exons exist for 45% of multi-exon loci, we sought to evaluate the gene structures that allowed alternative 5' exon usage and attempted to determine whether the predicted alternative starts could be verified by 5'-RACE (5' rapid amplification of cDNA ends). To evaluate the structure of variant 5' exon usage, we separated the set into three classes of alternative transcript (Figure 1): transcripts that start from mutually exclusive first exons; transcripts that originate from intronic regions of the genome and then continue on to the next exon; and transcripts that appear to initiate within coding exons of a longer canonical form. To demonstrate the relative frequency of each class we focused only on those loci with 50 counts or more for both starting exons (Table 4). The majority of these alternative starts was due to mutually exclusive starting exons, and more than half of these were within the first intron. None of the examples with 50 counts or more started within coding exons of a longer canonical form; the best supported example of this was a clone of Fgfr2 that starts within the 11th exon of the canonical form and is supported by 48 tags (GenBank:AK081810).

To test whether the threshold of counts we applied was biologically relevant and whether cDNAs starting from within internal exons of longer transcripts are 5' truncations or genuine transcription start sites, we tested a panel of 19 alternative 5' exons with 5'-RACE. As a technical point, an enzymatic oligo-cap method independent of the FANTOM3 cap-trapper technique was used to ensure that only full-length capped 5' ends of mRNAs were surveyed [29,30]. Predicted alternative 5' exons were confirmed for all classes tested. Additionally, and perhaps surprisingly, transcript starts with counts below five were validated including alternative transcripts with only one cDNA as evidence (Acvr1c [GenBank:AK049089] and Ptprg [GenBank:AK144283]). The results of the 5'-RACE analysis and the primer sequences used are provided in Additional data file 5.

### Alternative peptides and domain structures

The analyses described above used all available cDNA evidence, with many variants only detected as partial EST sequences. Although ESTs provide a deeper sampling of alternative transcripts, interpretation of variants found in these sequences is confounded by their bias to the termini of transcripts (due to EST sequence generation providing short reads coming from 5' and 3' termini of cDNAs) and problems associated with sequence quality arising from single sequencing reads for each EST. We therefore chose a more conservative approach and used only full-length cDNAs to examine alternative peptides encoded from these loci.

A total of 5,877 phosphoregulator full-length transcripts from FANTOM, GenBank, and RefSeq were filtered based on the following: redundant entries that shared the same splice junctions, TSS, and TTS were removed; transcripts with stop codons more than 50 bases upstream of their final splice junction were excluded as NMD candidates [10] (Additional data file 8); and transcripts with 5' or 3' truncated ORFs were removed. This left a core set of 639 loci with 2,358 transcripts that were predicted to encode 1,469 full-length peptides (Table 5).

**Figure 2:**
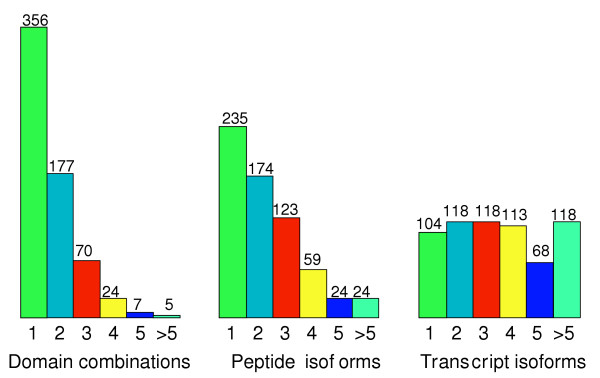
Relationship between transcript isoforms, peptide isoforms, and domain combinations.

The domain structure of these 1,469 peptides was then reviewed using InterProScan domain predictions [21]. Using these predictions we identified 1,080 unique combinations of domains and locus. Figure 2 summarizes the number of variant transcripts, peptides, and domain combinations observed within the phosphoregulator set. A major feature of this figure is the disparity between the number of alternative transcripts and alternative peptides. Eighty-four per cent of loci are identified as having multiple transcript isoforms, whereas 63% of loci have multiple peptides and only 44% have multiple domain combinations.

In a further analysis we compared the domain content of the 1,080 domain combinations with the domain complements of each locus (that is, the set of predicted domains from all transcripts of a given locus). Variant peptides were then classified into the following four classes: 582 peptides with the full complement; 147 variants with disrupted or missing accessory domains; 161 variants with disrupted or missing catalytic domains; and 190 with disruptions to both accessory and catalytic domains (Additional data files 9 and 11). These classifications were then added as annotations in the web interface. A list of all variants detected is provided in Additional data file 11. In Tables 6 and 7 we highlight two subsets of interest: 18 noncatalytic variants that maintain the full set of accessory domains, and 25 catalytic variants that remove all accessory domains. The accessory domains lost from these catalytic variants are largely interaction domains (PDZ, SH2, doublecortin, PKC PE/DAG, pleckstrin homology). The role of variants consisting only of accessory domains is unknown.

### Alternative forms of the receptor kinases and phosphatases

A class of phosphoregulators with multiple reported examples of transcriptionally derived dominant negative products is the receptor kinases. For these loci, multiple soluble secreted and membrane-tethered decoy receptors lacking catalytic domains have been described. We therefore undertook a computational review of transcripts of the 56 tyrosine receptor kinase, 12 serine/threonine receptor kinase, and 21 tyrosine receptor phosphatase loci of mouse to determine their potential to generate dominant negative gene products.

Conceptually, receptors are divided into two parts: the extracellular ligand-binding portion of the peptide and the intracellular catalytic portion. Signal peptide and transmembrane domains are both required for correct targeting and anchoring of type I membrane peptides within the plasma membrane. Each transcript variant was reviewed for changes in the predicted peptide that would affect localization signals or catalytic domains.

We identified two classes of ORFs encoding catalytically inactive variant peptides predicted to compete for ligand in the extracellular space (Table 8): 13 potential tethered decoys possessing intact transmembrane and extracellular domains, of which four had been reported previously in the literature; and 26 potential soluble secreted proteins possessing the ligand-binding domain and no transmembrane domain, of which seven had previously been reported.

The review of these loci also identified a further two classes of potential variants. Alternative TSS within loci frequently generated transcripts encoding peptides that lacked amino-terminal features. Many of these variants lacked the signal peptide (*n *= 13), whereas others lacked both the signal peptide and the transmembrane domain (*n *= 12). We refer to these two variant types as 'TMcatalytic' and 'catalytic', respectively. TMcatalytic forms resemble the type 2 transmembrane phosphoregulators such as the nonreceptor phosphatase Ptpn5, which localizes to the endoplasmic reticulum [31], and the kinase Nok, which localizes to cytoplasmic puncta [32]. We identified 13 of the TMcatalytic class and 12 of the catalytic class (Table 8).

We then compiled supporting evidence for expression of these transcripts in normal mouse tissues (Additional data file 7). All but two of the secreted and tethered forms are generated by alternative 3' ends hence we searched for microarray probes and MPSS (massively parallel signature sequencing) signatures diagnostic of these alternative 3' ends. The Mouse Transcriptome Project (trans-NIH with Lynx MPSS™ technology) provides MPSS gene expression data from a panel of 85 tissue samples [33,34]. Similarly, the GNF (Genomics Institute of the Novartis Research Foundation) gene atlas provides gene expression data using Affymetrix arrays for a panel of 61 normal mouse tissues [35,36]. The Mouse Transcriptome Project provided support for nine of the secreted proteins, four tethered decoys, and one cytoplasmic catalytic form. The GNF gene atlas provided support for an additional four secreted and one tethered form.

MPSS also provided evidence for tissue-specific expression of nine novel isoforms: seven secreted forms (Epha1 in bladder, Epha7 in brain, Flt3 in spinal cord, Ptprd in hypothalamus, Ptprg in brain, eye, white fat, and lung, Ptpro in brain, and Ptprs in thalamus); one tethered form of Axl in kidney; and one catalytic form of Ptprg in brain, kidney, white fat, and cartilage. Similarly, the GNF gene atlas provided evidence for tissue-specific expression of two novel secreted isoforms: Ptprk in blastocysts and Ptprg in brain. For the catalytic and TMcatalytic forms of Ptpre and Ptpro, CAGE tags confirmed their reported restriction to the macrophage lineage [37,38].

**Figure 3:**
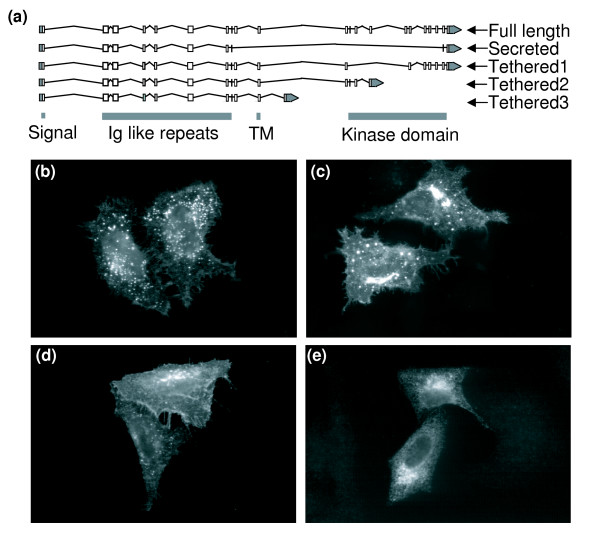
Alternative splice forms of the Csf1 receptor (c-fms). **(a) **Genomic alignment (mm5; chr18:61616977..61647364) of full-length and variant receptors displaying exon structure and peptide features. Also shown are subcellular localizations of variant receptors transiently expressed in HeLa cells: **(b) **full-length Csf1r (GenBank:AK076215); **(c) **Tethered1 (GenBank:AK155565); **(d) **Tethered3 (GenBank:AK171543); and **(e) **Secreted (GenBank:AK171241). Tethered forms are produced by exon skipping (Tethered1; c), termination within an intron (Tethered2), and a mutually exclusive alternative 3' exon (Tethered3; d). Tethered forms 1 and 3 exhibit similar localizations to that of the full-length receptor (panel b; cell surface and perinuclear puncta). The form lacking the transmembrane (TM) domain is absent from the cell surface and displays a secretory pathway-like localization.

As part of this review, we identified four novel transcripts for the colony stimulating factor 1 receptor Csfr1. Three of these transcripts were predicted to encode potential tethered isoforms, whereas a fourth encoded a potential secreted version of the receptor (Figure 3a).

In order to determine the likelihood of efficient expression and subcellular targeting of these novel variants, we undertook transient expression assays of the Csf1r variants in mammalian cells and confirmed that the truncated tethered forms are targeted, as predicted, to the plasma membrane whereas the form lacking the predicted transmembrane domain exhibits a secretory pathway-like localization (Figure 3).

**Figure 4:**
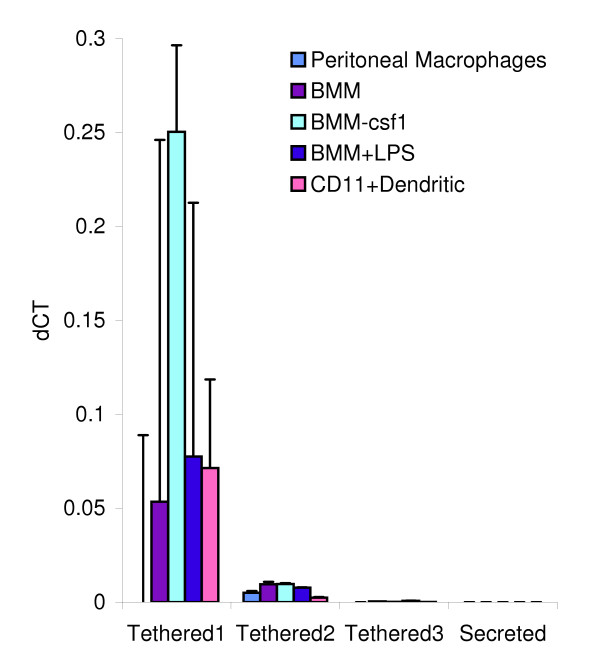
Expression of variant Csf1r transcripts relative to the full-length isoform. BMM, bone marrow derived macrophages; dCT, differences in cycle numbers between variant and full-length isoforms; LPS, lipopolysaccharide.

Finally, we sought to monitor the expression of all coding transcripts from the Csf1r locus to determine whether these transcripts are expressed at biologically relevant levels. Csf1r is known to be expressed in cells of the macrophage and dendritic lineages [39], and the three of the variants we identified as cDNAs were derived from CD11c-positive dendritic cells (two from the NOD mouse strain and one from C57BL/6J). Isoform-specific quantitative reverse transcriptase polymerase chain reaction (RT-PCR) for each variant was performed on a panel of CD11c-positive dendritic cells, peritoneal macrophages, and bone marrow derived macrophages from black 6 mice. All three tethered forms were detected in dendritic cells and bone marrow derived macrophages, but only tethered form 1 (GenBank:AK155565) was detected at levels similar to those of the full-length receptor (Figure 4 and Additional data file 12).

## Discussion

In this report we focused on a computational review of transcriptional complexity in the protein kinase and phosphatase loci of mouse and on the impact of transcript diversity on the probable function of the variant peptides they encode. We found that 75% of phosphoregulator loci have alternative splice forms with multiple sequences as evidence that ranks these loci close to the 80% level of zinc finger proteins in terms of transcriptional complexity. A large amount of this complexity is generated by the use of alternative 5' and 3' exons, and we found that 45% of multi-exon loci had well supported alternative 5' exons. These estimates were made using all available mouse transcript evidence, but deeper sampling of the transcriptome would probably increase these estimates further.

### Functional relevance of variant transcripts

A number of workers have reported estimates of transcript diversity based on EST evidence [4-6,40]. To address the functional relevance of alternative transcripts detected as partial EST sequence, workers have used counts of independent ESTs and conservation between species as computational filters for artefacts. Conservation is likely to identify biologically valid splice variants, but lack of conservation cannot be assumed to mean that a variant is artefact. One paper reported that 14-53% of alternative junctions in human are not conserved in mouse [41], whereas in a more extreme example it was reported that only 10% in a set of 19,156 human loci have a conserved alternative splice junction in mouse [42]. Currently, the limited depth of transcript sequencing in both mouse and human makes it difficult to determine the true level of conserved alternative transcripts. As more high-throughput transcriptome sequence becomes available it will be important to address the number of variants in humans and their conservation in mouse.

Another estimate of functional relevance is to examine expression and tissue specificity of the transcript isoforms. Some authors have attempted to use EST evidence to assess expression levels and tissue specificity of isoforms [43,44]. For tissue specificity and cross-species conservation analyses, EST sequences are confounded by the problems of limited depth of sequence, tissue sampling, and quality of annotations. In this report we mined the mouse transcriptome project MPSS signatures and the GNF gene expression atlas probes to provide supporting evidence for 19 of the variant receptors identified. However, a deeper sequence sampling with new technologies such as splice junction arrays and libraries enriched for alternative transcripts will be needed if we are to address expression of variants at a transcriptome wide level [45,46].

These technologies will be needed to address a number of important questions. Are the variant transcripts expressed at biologically relevant levels or is there a certain level of biologic noise in the transcriptional machinery? Do variant transcripts from the same locus exhibit tissue restricted patterns distinct from other isoforms, or are they coexpressed? Are variants inducible or constitutively expressed?

### Functional diversity of variant receptor kinases and phosphatases

In the case of receptor kinases and phosphatases, dominant negative forms that are capable of competing for ligand and downregulating signal transduction were previously reported (sFlt1 [47], Erbb2 [48], Epha7 [49], and Ntrk2 [50]). Mechanistically, cells expressing a tethered decoy would be predicted to fail to respond to ligand, whereas secreted forms have the potential to dampen the response in multiple cells by competing for ligand. Among the receptors we identified, 26 were putative secreted forms, of which 19 were novel to any species, and 13 were tethered forms, of which nine were novel. For example, we identified four catalytically inactive colony stimulating factor 1 receptor (Csf1r) variants in mouse, three of which were membrane associated whereas the fourth, lacking the transmembrane domain, appeared to localize to the secretory pathway (Figure 3). While we were preparing this paper, a report describing a soluble secreted form of Csf1r in goldfish showed that the peptide was detectable in fish serum and produced by macrophages, and was able to inhibit macrophage proliferation *in vitro *[51].

We also reported probable dominant negative forms for eight of the 14 Eph receptors in mouse (Epha1, 3, 4, 5, 6, 7 and 10, and EphB1) and a review of sequences from other species revealed probable dominant negative forms for three of the remaining six (EphB2 [52], secreted Epha8 [GenBank:NM_001006943, GenBank:BC072417], and tethered EphB4 [GenBank:AB209644]). A role for these variants in cell migration is supported by observations for Epha7 variants and the catalytically inactive Ephb6 [18,49]. Cells expressing tethered Epha7 variants exhibit suppressed tyrosine phosphorylation of the full-length form and altered migration behaviour to adhesion instead of repulsion toward ephrin-A5 ligand expressing cells [49].

Other tyrosine receptor kinase families enriched with probable dominant negative variants were the Vegf receptor family (Flt1, Flt3, Kdr, and Pdgfra) and the insulin receptor related genes (Alk, Insrr, and Insr). Alternative splicing of exon 11 of the insulin receptor in human has previously been reported [53], but no native secreted splice forms have yet been described.

Proteolytic processing for many of these receptors split the protein into a soluble extracellular fragment that is capable of binding ligand and an intracellular catalytic fragment (Erbb4 [54], Fgfr1 [55], and Tie2 [56]). The alternative transcripts we describe here are likely to mimic these forms and have similar activities, but the use of alternative transcription provides an independent mechanism of control in generating these products.

### Assessing the impact of variant domain structures

By using the concept of a domain complement for each locus we identified variants with alternative catalytic potential or changes in accessory domains. Most of the accessory domains are targeting, regulatory, or interaction domains. Two loci that we highlight in Tables 6 and 7 and in Additional data file 2 are Araf and Dcamkl1. In both cases, noncatalytic peptide forms consisting of only the accessory domains are produced by the use of alternative 3' ends. The Dcamkl1 locus uses both alternative promoters and terminators to generate three major forms, each with different predicted activities and localizations: the full length peptide targeted to the microtubules by the doublecortin domain; a form lacking the catalytic domain; and a form lacking the doublecortin domain [57] that resembles the active fragment released from microtubules on proteolytic cleavage by calpain [58]. Although the identification of an alternative 3' end in Araf may explain the two protein isoforms detected in mitochondria [59], the role of a noncatalytic isoform consisting of the Ras binding domain (InterPro:IPR003116) and the protein kinase C phorbol ester/DAG binding domain (InterPro:IPR002219) is unknown. Similarly, the role played by a noncatalytic form of Dcamkl1 consisting of only the microtubule associating doublecortin domain (InterPro:IPR003533) is unknown. A likely possibility is that these forms compete with the full-length version for associations with third party interactors.

### Other variants

A number of other variant transcripts occur within the phosphoregulator loci. Alternative splicing of mutually exclusive exons within the catalytic domain of Mapk14 (p38 and CSBP1/2) [60] are known to affect activity and substrate specificity. Variants of the related kinases Mapk9 and Mapk10 also appear to use mutually exclusive exons within the catalytic domain.

Another class of variant transcripts is predicted to undergo NMD. Using the '50 base rule', transcripts with premature termination codons more than 50 bases upstream of a final exon junction were filtered out as NMD candidates targeted for destruction [10]. However, NMD candidates may still represent a functional output of a locus. Recently, the term RUST (regulated unproductive splicing and translation) has been coined to describe the use of unproductive splicing to regulate protein expression [61].

Despite this, a number of the transcripts that break the 50 base rule still appear to represent full length messages with short predicted introns in their 3' untranslated regions. We identify four loci Rps6ka4, Map3k1, Epha4, and Pxk that have predicted final introns in their 3' untranslated regions of 126, 1555, 3239, and 114 bases, respectively. All NMD predictions are provided in Additional data file 8 and online [27].

### Peptide variants represent additional components of the system

In cases in which peptide variations disrupt or remove an accessory domain, constitutively active [62-64] or dominant negative [65] forms may be generated. Similarly, peptides with disruptions to the catalytic domain have been recorded as dominant negative forms (for example, Mask [66] and Mapk7 [67]). In loci such as Dcamkl1, which contain a targeting domain, the subcellular localization of the peptide can be changed and may allow access to different pools of substrate [57].

These variants not only add to the peptide diversity of the phosphorylation system, but they are also intrinsically related to the function of all peptides generated from the same locus. They are likely to compete for the same ligands and substrates, but by changes in the peptide their activity, stability, localization, and regulation may be altered. This opens up the possibility that transcriptional control of the mix of isoforms present within a system is used as an additional mechanism to regulate the overall status of the system.

### Transcriptional control

Regulated use of alternative promoters, terminators, and splice junctions allows a cell to produce either alternative peptides with slightly different activities or the same peptide in a different context. In some cases these choices are 'hard wired' during differentiation, such that one isoform is produced in a particular cell type (for example, fibroblast growth factor receptor splice variants in mesenchyme and epithelium [19]) whereas in others the changes are inducible (for example, Prkcb isoforms on insulin treatment [68]). In the case of the inducible changes there is evidence for a coupling of signal transduction to transcript isoform. For Prkcb, the inclusion of the PKC-betaII exon, within 15 minutes of insulin treatment, has been shown to be via activation of Akt signaling and phosphorylation of SRp40 [69]. Phosphorylation of transcription factors, spliceosome components, Histone H3, and the carboxyl-terminal domain of RNA polymerase all point to a closer role for phosphorylation in regulation of transcript isoform [70-73].

## Conclusion

Systematic analysis of every protein kinase and phosphatase of mouse has revealed that for most of these loci alternative transcripts are generated. The use of alternative transcription initiation, termination, and splice junction sites offers three mechanisms for controlling the functional output of the locus. We provide evidence for alternative 5' and 3' end usage and document a large set of variant peptides and domain structures. Finally, we suggest that, for complete understanding of signal transduction and protein phosphorylation in general, these forms must be considered components of the network and that regulation of these forms in development and on challenge indicates a fundamental coupling of transcriptional control with protein phosphorylation.

## Materials and methods

### Locus based visualization of phosphoregulators

For each locus a three frame view combined genomic and transcript centric views from FANTOM3 [20,74] with a summary table used to navigate between variant transcripts (Additional data file 2). The summary table provides Isoform transcript and peptide identifiers, representative nucleotide accession number, coding potential, InterPro predictions, 5' and 3' support, and NMD predictions. The comments field gives a simple description of how the transcript differs from other forms. The genomic view is provided by FANTOM3 and is an implementation of the generic genome browser [75]. Additional features mapped to the genome include InterPro predictions [21] and GNF symatlas expression data probes [36]. Mapping of peptide features was carried out in two parts. First, the nucleotide coordinates of the feature relative to the transcript were determined; these were then transposed to their genomic locations based on transcript to genome alignments provided by FANTOM3 [20,76]. The interface and custom GFF tracks are available online [27]. Nucleotide accession numbers for each locus are provided online and can be queried by Mouse Genome Database locus and synonyms [77,78].

### Mapping of transcript 5' and 3' ends

The 5' and 3' ends of full-length cDNA, ESTs, and tag sequences from CAGE [25], Genomic Sciences Center DiTags [20], and gene identification signature DiTags [26] were used to provide supporting evidence for alternative 5' and 3' ends.

Conceptually, two levels of clustering were carried out to provide end support. Tag clustering grouped transcripts that shared TSS or TTS based on the overlap of their termini (20 bases) relative to the UCSC mm5 (*Mus musculus *5, Mouse genome assembly, build 33) assembly of the mouse genome sequence (May 2004) [76]. Exon clusters grouped transcripts that shared the same first donor site or final acceptor site for 5' and 3' exon clusters, respectively.

### Exon junction clustering

The genomic mappings of every multi-exon cDNA and EST were extracted from the FANTOM3 analysis [20,76]. Exon junction support was provided by a count of the number of sequences that shared the same splice combination. Low quality alignments were filtered out by removal of exons mapping to the genome with under 99% sequence identity.

### Tissue specific expression of receptor isoforms

The nucleotide sequences of the probes used in the GNF gene atlas arrays and the MPSS signature sequences were aligned to transcript sequences using BLAST (basic local alignment search tool) [33,35]. Diagnostic probes were defined as probes that matched only the variant transcript isoform and had a perfect match for the entire length of the probe.

### Nonsense mediated decay

NMD predictions were made by calculating the distance between the last splice site and the stop codon of full length predicted. Splice sites were determined by alignments to mm5. A total of 191 sequences for which the final splice site was more than 50 bases from the stop codon were flagged as putative NMD targets [10]. A number of the final splice sites were suspected as artefactual alignments with very short predicted intron lengths. To remove these artefacts a further requirement was imposed that the minimum intron length had to be greater than 80 bases. This reduced the set to 120 predicted NMD candidates. These predictions were reviewed manually. All NMD predictions are provided in Additional data file 8 and online [27].

### Feature based assessment of protein function

For each locus, InterProscan predictions were used to assess changes in domain content of each variant [21]. Using this we determined the domain content for each full length transcript and then used predictions for every transcript to determine the domain complement for each locus. Domain changes were assessed by comparing the domain content of the predicted peptide with the domain complement of the locus. Additionally, for the receptor set, TMHMM and signalP predictions were used to detect transmembrane domains and signal peptides [79,80]. Variant receptors that lacked the transmembrane domain but retained the signal peptide were classified as probably secreted decoy receptors, whereas transmembrane forms lacking the catalytic domain were classified as probably tethered decoys.

### Subcellular localization of Csf1r (c-fms) variant transcripts

cDNA clones of variant Csf1 receptor (DDBJ:AK171241, DDBJ: AK155565, DDBJ:AK171543, and DDBJ:AK146069) were subcloned into a mammalian expression vector. HeLa cells were transiently transfected for 16 hours, formalin fixed, and processed for immunofluorescence. Recombinant Csf1r was detected using the rat monoclonal AFS98 antibody [81].

### Validation of Csf1r (c-fms) variant transcripts

RNA was harvested from cells using the RNeasy kit (Qiagen, Melbourne, VIC, Australia). First strand synthesis was carried out on 1 μg total RNA using Superscript III (Invitrogen, Melbourne, VIC, Australia). Real-time PCR was performed with the SYBR qPCR SuperMix-UDG kit (Invitrogen). Twenty microliter reactions were performed in an ABI 7700 (Applied Biosystems, Melbourne, VIC, Australia), with 35 cycles of 1 minute elongation at 60°C; all reactions were performed in duplicate. Relative fold change of full length and variant were calculated using the delta Ct (cycle threshold) method.

### 5'-RACE experiments

5'-RACE experiments were performed using an enzymatic oligo-capping method [29] that ensures capture of full-length capped 5' ends (Generacer; Invitrogen). Reverse transcription using random hexamers was carried out to generate 12 libraries from six tissues (total RNA if possible from male and female mice was mixed for the following tissues: whole body embryo day 10 [e10d], whole body embryo day 17.5 [e17.5d], adult whole brain [brain], adult testis [testis], neonate 2 days thymus [neo2d_thymus], and adult liver [liver]). Nested primers running back towards the 5' ends of the transcripts were then used in conjunction with a primer against the 5' ligated oligo to amplify the 5' ends of these cDNAs. The PCR products were then cloned into the pCR4-TOPO vector and 24 colonies from each library sequenced. The resulting sequences were then aligned to the genome by BLAT and the mappings are available as an optional GFF track in the genome viewer (these are provided with the primer sequences in the Additional data file 5).

## Additional data files

The following additional data files are available (and also on the associated website [27]): an Excel file listing all protein kinase-like and protein phosphatase-like loci considered in this study (sheet 1 lists the 522 kinase-like and 158 phosphatase-like loci with detected transcripts; sheets 2 and 3 provide details of the entries retired because of false positives, and duplications in reported by Forrest [22] and Caenepeel [23] and their coworkers; and sheet 4 provides a list of predicted transcripts still awaiting confirmation by cDNA evidence; Additional data file 1); a pdf file containing a pair of screen captures demonstrating visualization of the Araf and Dcamkl1 protein kinase loci (note alternative well supported 5' and 3' exons that structurally divide the loci; Additional data file 2); Excel file listing alternative splice junctions identified in the set and the cDNA accession numbers that support them (Additional data file 3); a zip file containing four Excel files (5' exon, 3' exon, TSS and TTS clusters; Additional data file 4); a zip file containing a PowerPoint presentation with genomic views of the 5'-RACE results and an Excel file summarizing the results and the primer sequences used (Additional data file 5); an Excel file of zinc finger loci with levels of support for alternative transcripts (Additional data file 6); an Excel file that contains supporting evidence for the variant receptors discussed in the results, providing links to MPSS, GNF, and CAGE for transcriptional evidence, links into PubMed for known examples, and other supporting evidence (Additional data file 7); a pdf file containing a listing of clones predicted as NMD candidates (Additional data file 8); an Excel file containing the domain combinations, complements, and raw Interpro results for all full-length transcripts in the phosphoregulator set (Additional data file 9); a pdf file showing a graph of the number of loci with alternative splice junctions, and 5' terminal or 3' terminal exons (for a junction to be considered variant it requires two independent cDNAs - one cDNA flags the sequence as potential; for terminal exons a count of five events is required for it to be considered variant - two events flag the sequence as potential; Additional data file 10); an Excel file summarizing the predicted domain combination and variant type for the 1473 full-length ORFs identified in the domain structure analysis (Additional data file 11); a zip file containing an Excel file summarizing the quantitative real-time PCR results for the Csf1r receptor variants and a pdf file containing additional localization images for the secreted isoform (Additional data file 12).

## Supplementary Material

Additional data file 1 An Excel file listing all protein kinase-like and protein phosphatase-like loci considered in this study (sheet 1 lists the 522 kinase-like and 158 phosphatase-like loci with detected transcripts; sheets 2 and 3 provide details of the entries retired because of false positives, and duplications in reported by Forrest [22] and Caenepeel [23] and their coworkers; and sheet 4 provides a list of predicted transcripts still awaiting confirmation by cDNA evidence). Click here for file

Additional data file 2 A pdf file containing a pair of screen captures demonstrating visualization of the Araf and Dcamkl1 protein kinase loci (note alternative well supported 5' and 3' exons that structurally divide the loci). Click here for file

Additional data file 3 An Excel file listing alternative splice junctions identified in the set and the cDNA accession numbers that support them. Click here for file

Additional data file 4 A zip file containing four Excel files (5' exon, 3' exon, TSS and TTS clusters). Click here for file

Additional data file 5 A zip file containing a PowerPoint presentation with genomic views of the 5'-RACE results and an Excel file summarizing the results and the primer sequences used. Click here for file

Additional data file 6 An Excel file of zinc finger loci with levels of support for alternative transcripts. Click here for file

Additional data file 7 An Excel file that contains supporting evidence for the variant receptors discussed in the results, providing links to MPSS, GNF, and CAGE for transcriptional evidence, links into PubMed for known examples, and other supporting evidence. Click here for file

Additional data file 8 A pdf file containing a listing of clones predicted as NMD candidates. Click here for file

Additional data file 9 An Excel file containing the domain combinations, complements, and raw Interpro results for all full-length transcripts in the phosphoregulator set. Click here for file

Additional data file 10 A pdf file showing a graph of the number of loci with alternative splice junctions, and 5' terminal or 3' terminal exons (for a junction to be considered variant it requires two independent cDNAs - one cDNA flags the sequence as potential; for terminal exons a count of five events is required for it to be considered variant - two events flag the sequence as potential). Click here for file

Additional data file 11 An Excel file summarizing the predicted domain combination and variant type for the 1473 full-length ORFs identified in the domain structure analysis. Click here for file

Additional data file 12 A zip file containing an Excel file summarizing the quantitative real-time PCR results for the Csf1r receptor variants and a pdf file containing additional localization images for the secreted isoform. Click here for file

## Figures and Tables

**Table 1:** Protein kinase and phosphatase loci of mouse

Classification	*n *
Kinase-like	527
Phosphatase-like	160
Transcript evidence	
Observed transcript	680
Gene predictions	7
Gene architecture	
Multi-exon	679
Single exon	8
Total	687

**Table 2:** cDNA evidence

Transcript support	5' end	3' end
FANTOM3	3,211	3,211
PUBLIC	2,666	2,666
5' ESTs	20,866	-
3' ESTs	-	32,166
Public ESTs	41,543	15,989
GIS	1,279	1,279
GSC	27,616	27,616
CAGE	162,707	-
Total count	259,888	82,927

Breakdown of supporting transcript evidence used in the paper: full-length cDNAs (FANTOM3, public), expressed sequence tags (ESTs; public ESTs, and RIKEN 5' and 3' ESTs), capped analysis of gene expression (CAGE) tags, and DiTags (gene identification signature [GIS] and Genome Sciences Centre [GSC]).

**Table 3:** Support for alternative transcription starts and stops within the phosphoregulator set

End		5 counts	10 counts	20 counts	50 counts
5'	5' exon clusters	1086/612 (1.8)	852/576 (1.5)	730/543 (1.3)	577/480 (1.2)
	TSS clusters	1289/609 (2.1)	924/572 (1.6)	742/533 (1.4)	550/472 (1.2)
3'	3' exon clusters	976/611 (1.6)	750/564 (1.3)	576/495 (1.2)	335/307 (1.1)
	TTS clusters	1600/620 (2.6)	1054/566 (1.9)	685/483 (1.4)	307/262 (1.2)

Number of 5' or 3' ends are shown for thresholds of 5, 10, 20 or 50 supporting tags. Shows the number of ends divided by the number of genes, and the ratio in brackets Note that at a threshold of 50, the number of genes with 3' end support is almost half that with 5' support. TSS, transcription start site; TTS, transcription termination site.

**Table 4:** Loci with well supported alternative 5' exons

Intron	Type	Count	MGI symbol
1	ME_exon	16	Abl1, Adck1, Brd4, Dusp14, Mark2, Pak1, Pdp1, Pkn3, Prkacb, Prkar1a, Ptp4a3, Ptprs, Raf1, Riok2, Sgk, Srpk2
	Intronic	9	Acvrl1, Ccrk, Cdk9, Ntrk2, Pim3, Ppp4c, Prkcn, Prkwnk1
2	ME_exon	1	Sgk3
	Intronic	1	Ptp4a2
3-4	ME_exon	6	Mast3, Limk2, Pak6, Pftk1, Pkn1, Prkcz
	Intronic	0	
5>	ME_exon	6	Dcamkl1, Lats2, Plk1, Ptprd, Tns1, Tns3, Ttn
	Intronic	2	Mylk, Ptpro

The Intron column refers to the intron where alternative transcript begins, and the Count column shows the number of loci in each class. Intronic, starts in intron runs into next exon; ME_exon, mutually exclusive first exons.

**Table 5:** Breakdown of transcript and peptide sets used in the variant analyses

	Total set	Full-length cDNAs	Transcript isoforms	Peptide encoding transcripts	Peptide isoforms	Domain combinations
Loci	687	676	676	639	639	639
Variants	-	5,877	4,496	2,358	1,469	1,080

Unique transcripts and unique peptides were identified by the Isoform Transcript Set (ITS) and Isoform Peptide Set (IPS) sequences identified by Carninci and coworkers [20].

**Table 6:** Catalytic variants lacking all accessory domains

MGD symbol	Transcripts	Catalytic	Accessory domains removed
B230120H23Rik	AB049732	+	SAM, H^+ ^transporter IPR000194
Bmp2k	AK046752	+	IPR011051 RmlC-like cupin
Camk2d	AK032524	+	NTF2
Dcamkl1	AK032424	+	Doublecortin domain
Ddr2	AK132504	+	Ligand binding ectodomain
Irak2	AY162380	+	Death domain
Jak1	BC031297	+	SH2, Band4.1/Ferm
Map3k14	AF143094		omega toxin-like. (SSF57059)
Mapk8	AB005663	+	H^+ ^transporter IPR000194
Mast1	AK141034	+	PDZ domain (IPR001478).
Pik3r4	AK042361	+	ARM repeat fold, WD40 repeats and HEAT repeats.
Plk4	AK045082	+	C-terminal polo-box domain
Ppm1a	AF369981	+	SSF81601 Protein serine/threonine phosphatase 2C, C-terminal
Prkx	AK039088	+	Protein kinase c terminal domain(IPR000961)
Ptpn21	D83072	+	Band4.1/Ferm
Ptprb	AF157628	+	Ligand binding ectodomain
Ptprd	BC025145	+	Ligand binding ectodomain
Ptpre	U36758	+	Ligand binding ectodomain
Ptprg	AK144283	+	Ligand binding ectodomain
Ptprs	AK159320	+	Ligand binding ectodomain
Ptpro	U37466	+	Ligand binding ectodomain
Rps6kc1	BC058403	+	MIT, PX
Stk36	AK007188	+	ARM repeat fold
Tns1	AK053112	+	SH2 and pleckstrin homology/phosphotyrosine interaction domain
Zap70	AB083210	+	SH2

**Table 7:** Noncatalytic variants with the full set of accessory domains

MGD symbol	Transcripts	Catalytic	Accessory domains in noncatalytic form
Araf	AK133797	-	Ras-binding domain (IPR003116), PKC PE/DAG binding domain (IPR002219)
Camk2a	X87142	-	C-terminal SSF54427 domain
Cwf19l1	AK088543	-	CwfJ domain only
D10Ertd802e	AK139747	-	ARM repeat fold only
Dcamkl1	AK043874	-	Doublecortin domain
Dusp16	AK035652	-	Rhodanese domain only
Egfr	BC023729	-	Ligand binding ectodomain
Eif2ak3	AK010397	-	Quinonprotein alcohol dehydrogenase-like motif (IPR011047)
Ksr	AK164833	-	PKC PE/DAG (IPR002219)
Map2k5	BC013697	-	Octicosapeptide/Phox/Bem1p domain (IPR000270).
Map3k14	AK006468	-	Omega toxin-like (SSF57059)
Mark3	AK075742, BC026445	-	Ubiquitin associated domain and kinase associated c-terminal domain
Mast2	AK004728	-	PDZ
Mtm1	AK149997	-	Gram
Prkwnk1	BB619950	-	TONB box, site specific DNA methyltransferase
Ptpn14	AF170902	-	Band4.1/Ferm and Pleckstrin homology
Syk	AK036736		SH2
Tns1	AK004758	-	SH2 and pleckstrin homology/phosphotyrosine interaction domain

**Table 8:** Variant kinase and phosphatase receptor forms of mouse

Type	Loci	Novel	Known^a^
Secreted	Alk, Csf1r^a^, Egfr^ab^, Epha1^b^, Epha3^a^, Epha5, Epha7^b^, Epha10^a^, Ephb1, Flt1^ab^, Flt3^b^, Insr, Insrr, Kdr, Met, Ptk7, Ptprc, Ptprd^b^, Ptprg^b^, Ptprk^ab^, Ptprn, Ptprn2, Ptpro^b^, Ptprr, Ptprs^b^, Ptprz1^ab^	19	7
Tethered	Axl^b^, Bmpr1a, Csf1r, Epha4, Epha5, Epha6, Epha7^ab^, Ntrk2^ab^, Ntrk3^a^, Pdgfra^ab^, Ptprk, Ptprm, Ptpru	9	4
Tmcat	Axl, Ddr2, Epha6, Igf1r, Kit, Ntrk1, Ptprb, Ptpre^a^, Ptpro^a^, Ptprr^a^, Ptpru, Ror2, Tgfbr1	10	3
Catalytic	Acvr1c, Csf1r, Epha10, Fgfr1, Fgfr2, Kit^a^, Mertk, Ptpre^a^, Ptprg^b^, Ptprm, Ptpro^a^, Ptprs	9	3

^a^Previously reported variants [37,38,1,82-92]. ^b^Detected by massively parallel signature sequencing (MPSS) or Genomics Institute of the Novartis Research Foundation (GNF).
