# Supplementary material for: Genome-wide review of transcriptional complexity in mouse protein kinases and phosphatases
Source: Genome Biol. 2006 Jan 26;7(1):R5. doi: 10.1186/gb-2006-7-1-r5 (PMC1431701; doi:10.1186/gb-2006-7-1-r5)
Supplement: Additional data file 2 — A pdf file containing a pair of screen captures demonstrating visualization of the Araf and Dcamkl1 protein kinase loci (note alternative well supported 5' and 3' exons that structurally divide the loci). [file gb-2006-7-1-r5-S2.pdf]

Additional data file 2: Locus based visualization for every protein kinase and phosphatase of mouse (http://variant.imb.uq.edu.au/index.phtml?uid=330&h_feat=NULL&frames=3)
(Three frame view of the **Araf** locus, note alternative 3' ends that remove the kinase domain).
Top frame: Summary table of all unique full length transcripts identified for this locus (MGD locus name, ITS – identical transcript set ID, IPS – Identical peptide set ID, representative accession number, coding potential, interpro domain predictions, 5' and 3' support, Nonsense mediated decay prediction).
Centre frame: genomic visualization of transcripts from this locus (Red arrows at top display CAGE tags, alignment of full length cDNA sequences, CpG island, custom genomic mapping of summary Interpro domain predictions); Also available but not shown (EST, GIS and GSC alignments, Raw Interpro domain predictions, GNF-Affymetrix probe positions, Tissue specific CAGE tag clusters).
Bottom frame: Fantom3 transcript visualization. Transcript displayed is highlighted yellow in Top frame and centre frame. (Green – Translation, Red – DNA alignements, Blue – Peptide alignments, Pink – Domain predictions, Purple – SignalP and TMHMM predictions).

Results for Araf: raf-related oncogene(*araf, A-Raf*) - PREGID: 330 TKID: 836 Show Transcript: Yes No

VTS_ID	VPS_ID	Accession	Coding Potential	Domains	Comments	5' Exon Cluster Support	3' Exon Cluster Support	TSS Support	TTS Support	NMD
**TA836.1**	**PA836.1**	**BC004757**	**coding**	**IPR000719:IPR001245:IPR002219:IPR002290:IPR003116:IPR008271:IPR011009:PF07714.1:SSF54236:SSF57889**	**Full complement**	**422**	**29**	**422**	**29**	**no**
TA836.6	PA836.6	6820405M17	coding	IPR000719:IPR002219:IPR003116:IPR011009:SSF54236:SSF57889	Variant disrupts the catalytic domain	422	4	422	4	no
TA836.7	PA836.7	B230378I04	coding	IPR000719:IPR002219:IPR003116:IPR011009:SSF54236:SSF57889	Variant disrupts the catalytic domain	422	3	422	3	no
TA836.8	PA836.8	5730420C13	coding	IPR002219:IPR003116:SSF54236:SSF57889	Variant removes the catalytic domain	422	98	422	82	no
TA836.9	PA836.8	A230089M10	coding	IPR002219:IPR003116:SSF54236:SSF57889	Variant removes the catalytic domain	422	98	422	82	no

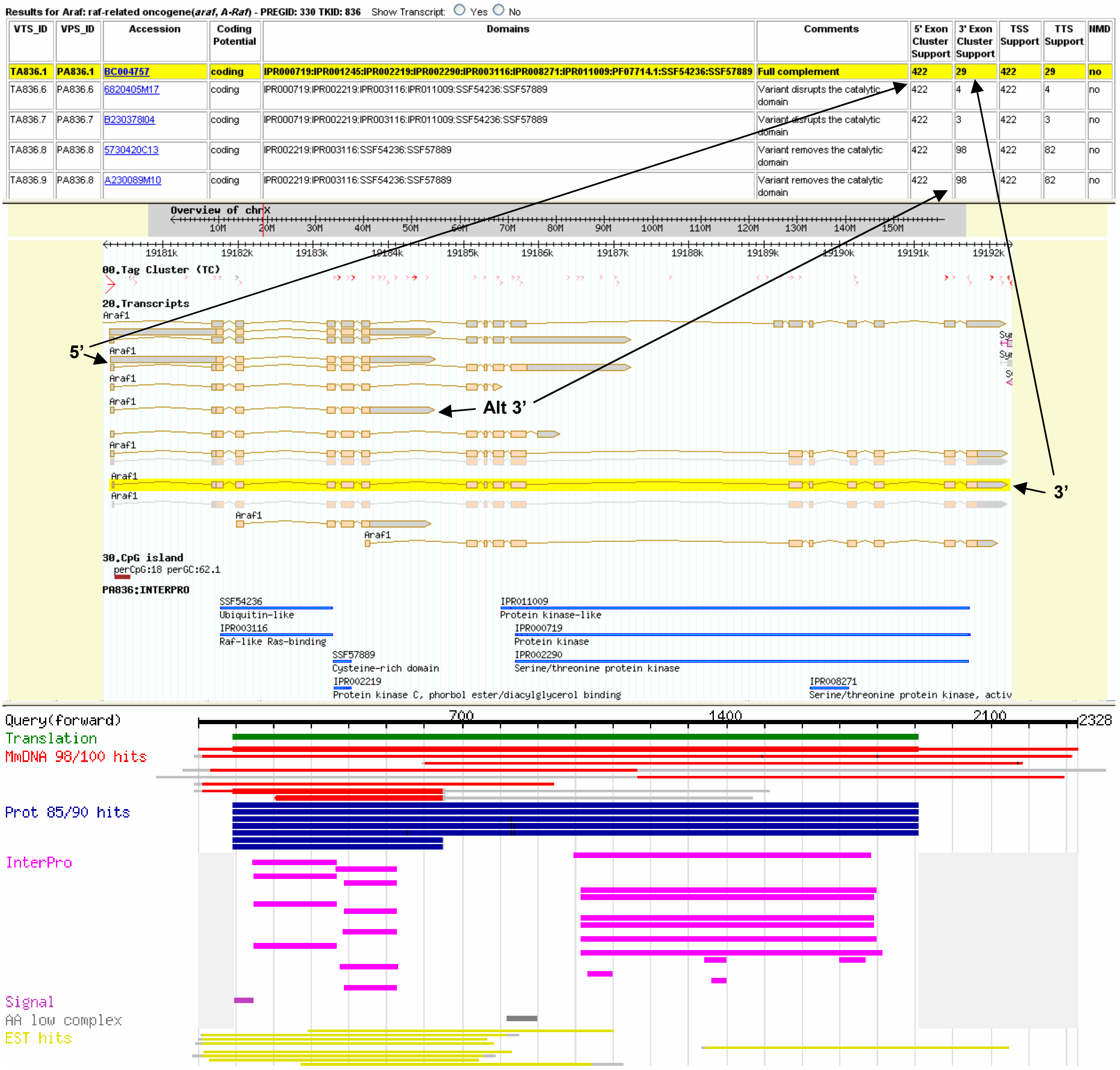

Results for Dcamkl1: double cortin and calcium/calmodulin-dependent protein kinase-like 1(*CPG16, DCLK, mKIAA0369*) - PREGID: 76 TKID: 222183 Show Transcript: ◯ Yes ◯ No

VTS_ID	VPS_ID	Accession	Coding Potential	Domains	Comments	5' Exon Cluster Support	3' Exon Cluster Support	TSS Support	TTS Support	NMD
TA222183.1	PA222183.1	AF155819	coding	IPR000719:IPR001245:IPR002290:IPR003533:IPR008271:IPR011009:SSF89837	Full complement	170	17	169	17	no
TA222183.5	PA222183.5	AF155820	coding	IPR000719:IPR002290:IPR003533:IPR011009:SSF89837	Variant disrupts the catalytic domain	170	17	169	17	no
TA222183.6	PA222183.6	C030016N17	coding	IPR000719:IPR001245:IPR002290:IPR008271:IPR011009	Variant removes all accessory domains	143	2	143	2	no
TA222183.7	PA222183.7	6430542M22	coding	IPR000719:IPR001245:IPR002290:IPR008271:IPR011009	Variant removes all accessory domains	143	49	143	45	no
TA222183.8	PA222183.8	AF155821	coding	IPR000719:IPR001245:IPR002290:IPR008271:IPR011009	Variant removes all accessory domains	143	49	143	45	no
TA222183.10	PA222183.10	A830044L14	coding	IPR003533:SSF89837	Variant removes the catalytic domain	170	4	169	4	no
**TA222183.33**	**PA222183.11**	**BC064783**	**coding**	**IPR003533:SSF89837**	**Variant removes the catalytic domain**	**170**	**105**	**169**	**37**	**no**
TA222183.36	PA222183.11	BC050903	coding	IPR003533:SSF89837	Variant removes the catalytic domain	170	105	169	59	no

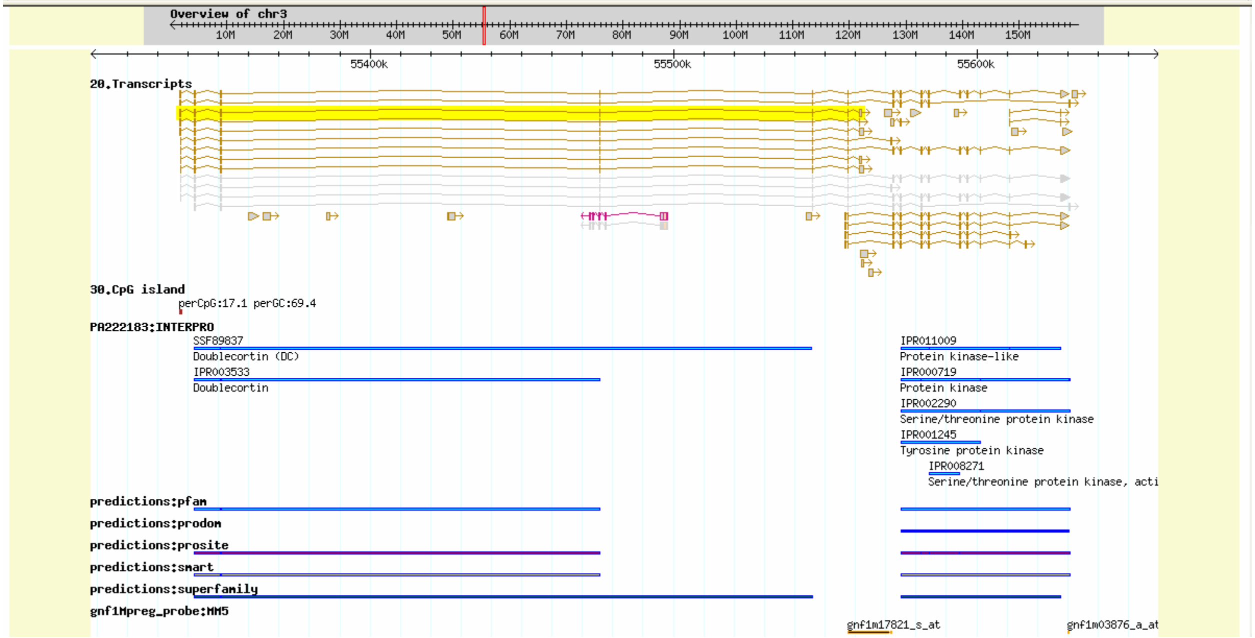

Visualization of the **Dcamkl1** locus (2 frame version).
(http://variant.imb.uq.edu.au/index.phtml?frames=2&uid=76&h_feat=NULL)
Note alternate well supported 5' and 3' exons that structurally divide the locus into two functional sections, the microtubule binding doublecortin domain and the catalytic kinase domain. Consensus Interpro predictions are mapped to the genome and displayed with the underlying member database predictions.
