# Supplementary material for: Genome-wide review of transcriptional complexity in mouse protein kinases and phosphatases
Source: Genome Biol. 2006 Jan 26;7(1):R5. doi: 10.1186/gb-2006-7-1-r5 (PMC1431701; doi:10.1186/gb-2006-7-1-r5)
Supplement: Additional data file 8 — A pdf file containing a listing of clones predicted as NMD candidates. [file gb-2006-7-1-r5-S8.pdf]

Alternate transcripts and the regulation of protein phophorylation systems.

Supplementary data to Forrest et al. 2005

Home - **NMD** - **Variant content** - **Receptors** - **Kinome** - **Phosphatome** - **Links**

Search for gene name, symbol or synonym (no wildcards required):

Submit Clear

This page lists nonsense mediated decay candidates:

NMD predictions were made by calculating the distance between the last splice site and the stop codon of full length predicted peptides from the IPS set. Splice sites were determined by alignments to mm5. 191 sequences where the final splice site was greater than 50bases from the stop codon were flagged as putative NMD targets (Hillman et al. 2004).

A number of the final splice sites were suspected as artefactual alignments with very short predicted intron lengths. To remove these artefacts a further requirement was made that the minimum intron length had to be greater than 80 bases. The predictions were then reviewed manually. This reduced the set to 120 predicted NMD candidates.

A number of transcripts still appear to represent full length messages with short predicted introns within the 3' UTR. We identify four such loci with predicted introns in their 3' UTRs 823, 318, 386 and 89 bases downstream of their respective stop codons.

1700091F14Rik(1700091F14), 2310004I24Rik(2310004I24), 2610019A05Rik(BC052063), 4932414J04Rik (4932414J04), 4932415A06Rik(A830013O05), A430105I05Rik(F730031B04), Acvr1(D330013D15), Acvr2 (E130303E12), Akt2(G430127A20), Als2cr2(AB057667), Als2cr2(B230377N01), B130003F20Rik(5031425M16), BC017634(BC006929), BC024265(BC024265), BC030499(A230108M20), Bmpr1b(B230317C16), Brd2(I920015J10), C230081A13Rik(C230081A13), Camk2g(E330023A07), Cdc14b(BC057357), Cdc7(AB018576), Cdc7(AB018577), Cdk2(U63337), Cdkl3(B130020N14), Clk(5930430M12), Clk(L29221), Clk(U21209), Clk3(9030616F20), Dapk1 (BC060161), Dm15(S60313), Dm15(S60315), Dusp12(AF280811), E130304F04Rik(BC051189), ENSMUSG00000044457(4930509O22), ENSMUSG00000047406(4932415M13), Ephb3(F930020L15), Ephb4 (E330033O04), Erbb2(BC053078), Erbb2(D030063B12), Ern2(A730094O08), Fgfr3(B230348E19), Frk(Z48757), Gprk6(Y15800), Gsk3a(2700086H06), Hipk1(AF071071), Ikbkb(F830015F03), Ikbkb(I830016M06), Irak1(AB088370), Irak2(9130227N12), Irak4(9330209D03), Jak3(L33768), Jak3(NM_010589), Kit(I0C0048O08), Limk1(F630024G05), Ltk(F830218H06), Mak(BC050009), Map2k3(BC007467), Map3k3(U43187), Map4k1(BC005433), Mapkapk5 (9930013M24), Mst1r(E130119A05), Mtmr3(BC032166), Mtmr4(BC058091), Mtmr7(BC032254), Nek1(S45828), Nek2 (I920184K11), Pak7(2900083L08), Pak7(6030481C12), Pctk2(BC031778), Pdgfra(9030623J19), Pdp1(9830138J19), Pik3r4(BC017537), Pip3ap(BC042573), Plk3(BC031180), Ppp1cb(E430038F13), Prkar1a(I830018G14), Prkce (A730046G04), Prpf4b(BC003769), Prpf4b(D130063E21), Ptk2(AB030035), Ptpla(D830025M12), Ptpn14(AF170902), Ptpn5(S80329), Ptprd(B530033M22), Ptprn2(A330094A06), Ptprs(D28531), Ptpru(BC048694), Ripk2(2210420D18), Rnasel(C130033H22), Rps6kb2(BC051631), Sgk3(F430203J07), Sgk3(NM_177547), Srpk2(BC062941), Stk11 (F830019B22), Stk36(D930047D01), Styx(0610039A20), Styx(U34973), Tie1(BC057004), Tpte(AJ311313), Trim28 (AF230392), Trrap(E330036C13), Ttbk1(AB046593), Vrk1(BC051072)

Potential full length transcripts that break the 50base rule
Epha4 (3239base intron, 823bases downstream of stop codon):
Epha4(4632410M06), Epha4(BC052164), Epha4(M5C1109J03), Epha4(S57168)

Map3k1 (1555base intron, 318 bases downstream of stop codon):
Map3k1(AF117340), Map3k1(L13103),
Pxk (114base intron, 386 bases downstream of stop codon):
Pxk(4632406H19), Pxk(BC016131), Pxk(C230080L11), Pxk(F830009F22), Pxk(F830208J01), Pxk(F830212K06), Pxk(G730030C19), Pxk(I920080F17),
Rps6ka4 (126base intron 89 bases downstream of stop codon):
Rps6ka4(BC012964), Rps6ka4(F730035M09)
