# Supplementary figures and images for: Genome-wide review of transcriptional complexity in mouse protein kinases and phosphatases
Source: Genome Biol. 2006 Jan 26;7(1):R5. doi: 10.1186/gb-2006-7-1-r5 (PMC1431701; doi:10.1186/gb-2006-7-1-r5)

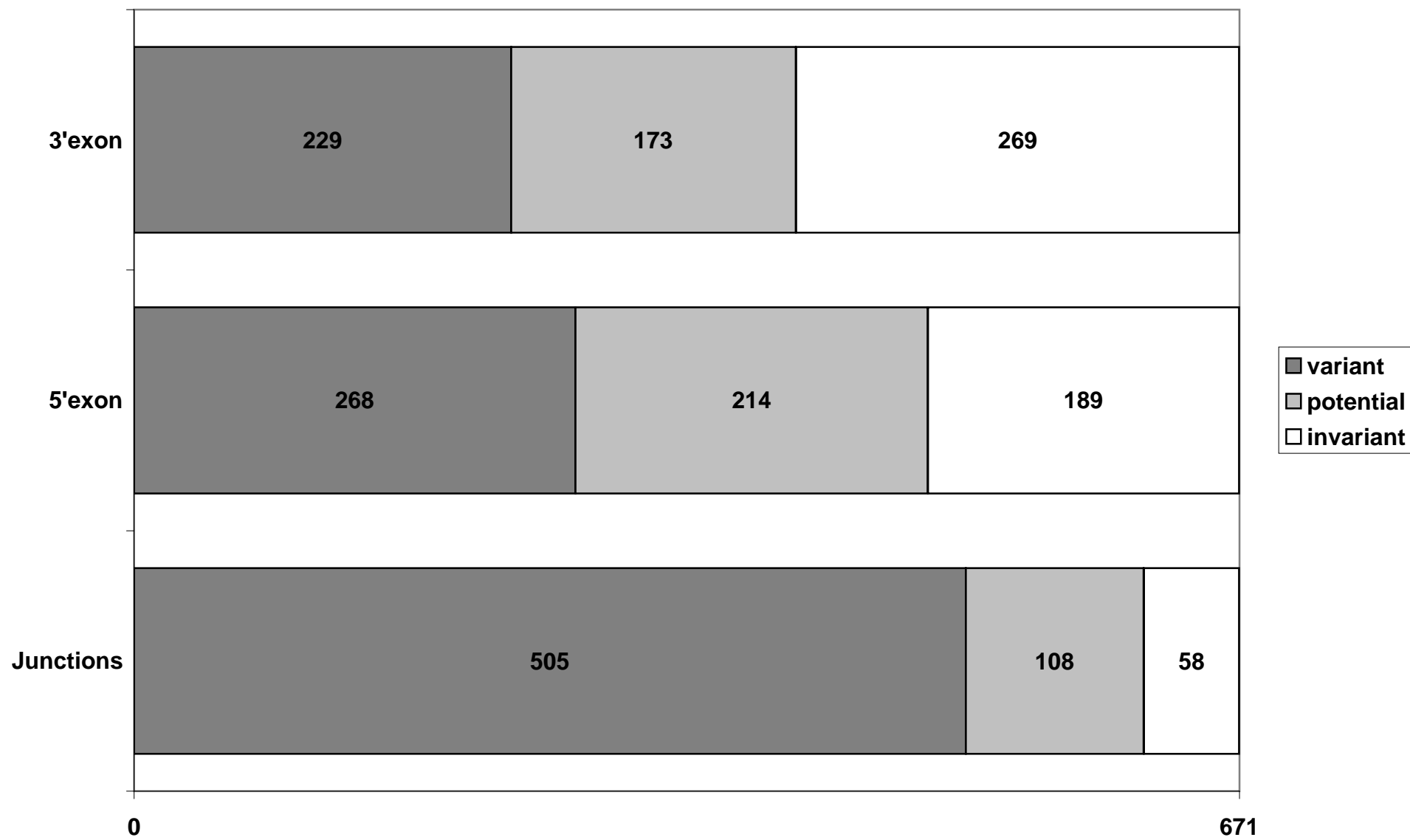

Supplement: Additional data file 10 — A pdf file showing a graph of the number of loci with alternative splice junctions, and 5' terminal or 3' terminal exons (for a junction to be considered variant it requires two independent cDNAs - one cDNA flags the sequence as potential; for terminal exons a count of five events is required for it to be considered variant - two events flag the sequence as potential). [file gb-2006-7-1-r5-S10.pdf]
