# Supplementary material for: Genome-wide review of transcriptional complexity in mouse protein kinases and phosphatases
Source: Genome Biol. 2006 Jan 26;7(1):R5. doi: 10.1186/gb-2006-7-1-r5 (PMC1431701; doi:10.1186/gb-2006-7-1-r5)

Additional images of the predicted secreted variant [GenBank:AK171241]

All transfected cells display a similar localization and lack cell surface staining.

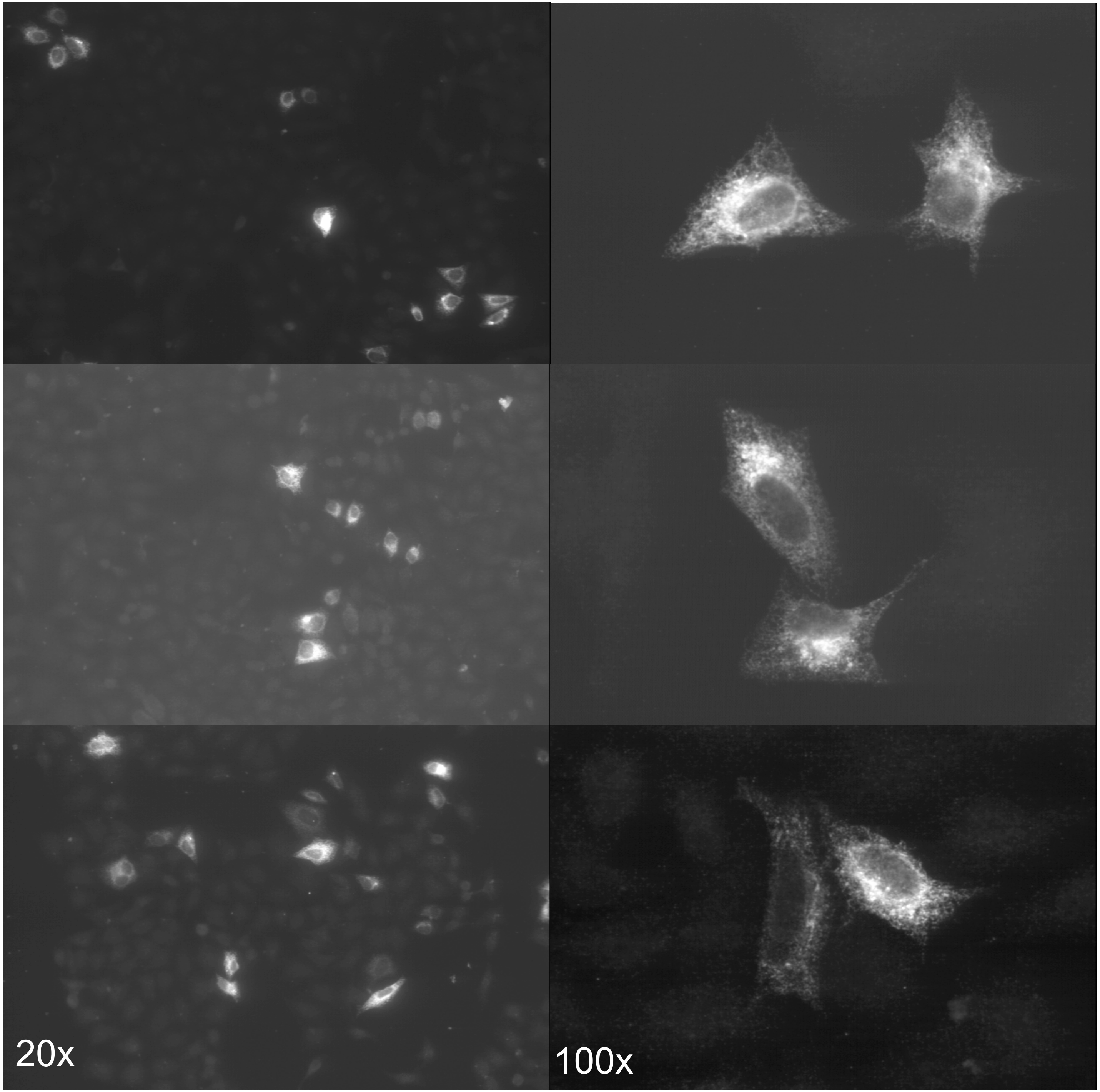

Supplement: Additional data file 12 — A zip file containing an Excel file summarizing the quantitative real-time PCR results for the Csf1r receptor variants and a pdf file containing additional localization images for the secreted isoform. [file gb-2006-7-1-r5-S12.zip › secreted.pdf]
